# Hydroxyapatite-coated implants provide better fixation in total knee arthroplasty. A meta-analysis of randomized controlled trials

**DOI:** 10.1371/journal.pone.0232378

**Published:** 2020-05-12

**Authors:** Tamara Horváth, Lilla Hanák, Péter Hegyi, Edina Butt, Margit Solymár, Ákos Szűcs, Orsolya Varga, Bui Quoc Thien, Zsolt Szakács, Endre Csonka, Petra Hartmann

**Affiliations:** 1 Institute of Surgical Research, University of Szeged, Szeged, Hungary; 2 Institute for Translational Medicine, Medical School, University of Pécs, Pécs, Hungary; 3 Department of Traumatology, University of Szeged, Szeged, Hungary; 4 Department of Surgery, University of Semmelweis, Budapest, Hungary; 5 Department of Preventive Medicine, Faculty of Public Health, University of Debrecen, Debrecen, Hungary; 6 Szentágothai Research Centre, Medical School, University of Pécs, Pécs, Hungary; Northeastern University, UNITED STATES

## Abstract

**Background:**

The potential advantages of hydroxyapatite (HA)-coated cementless total knee arthroplasty (TKA) implants are bone stock preservation and biological fixation. Studies comparing the outcomes of HA-coated cementless, non HA-coated cementless (uncemented) and cemented TKA implants reported contradictory data. Our aim was to provide a comparison of the effects of HA coating of tibial stem on the stability and functionality of TKA implants.

**Methods:**

A systematic literature search was performed using MEDLINE, Scopus, EMBASE and the CENTRAL databases up to May 31st, 2019. The primary outcome was Maximum Total Point Motion (MTPM) of the tibial stem. This parameter is determined by radiosterometric analysis and refers to the migration pattern of the prosthesis stems. The clinical outcomes of the implanted joints were evaluated by the Knee Society Knee Score (KSS) and the Knee Society Function Score (KFS). Weighted mean difference (WMD) with 95% confidence interval (CI) were calculated with the random-effects model.

**Results:**

Altogether, 11 randomized controlled trials (RCTs) with 902 patients for primary TKA implants were included. There was a statistically significant difference in the MTPM values with the use of HA-coated and uncoated uncemented implants (WMD = +0.28, CI: +0.01 to +0.56, *P*<0.001). However, HA-coated stems showed significantly higher migration when compared with the cemented prostheses (WMD = -0.29, CI: -0.41 to -0.16, *P*<0.001). The KSS values of HA-coated implants were significantly higher than those for the uncemented implants; moreover, KSS and KFS outcome scores were statistically not different between the HA-coated and cemented prosthesis cases.

**Conclusion:**

HA-coating yields better stability than other, uncemented prostheses. More importantly, the HA-coating is not outperformed by cemented prosthesis in providing good functional outcome.

## Introduction

Since the introduction of cementless prostheses, manufacturers came up with new materials with better biocompatibility and porous, rough surface to increase stability [1]. Among them, hydroxyapatite (HA) is a promising coating material with the potential to achieve biological fixation of implants [2]. In terms of its chemical structure, HA is an osteoconductive calcium phosphate molecule similar to human bone, which accelerates and induces insertion of implants, called osteointegration [2, 3]. Numerous studies investigated the outcomes of HA-coated stems with conflicting results. Some of them reported improved initial and late stability of stems, directly correlating with prosthetic life [3]. However, further investigations did not confirm these benefits and signs of osteolysis or early stem migration were observed [4].

Five previous systematic reviews and national registries have summarized the available evidences, but each of these has limitations [5–11]. Registries were based on observational data with potential sources of bias including the lack of worldwide consensus on implants taxonomy. Moreover, learning curve effects and differences between a high volume center and the wide community practice were also not explicitly addressed in these tables [12]. One systematic review [7] failed to eliminate potential selection bias because a few of the studies enrolled hybrid fixation (such as cemented femoral and uncemented tibial stem). Others included quasi-randomized and observational studies as well [9]. Two reviews allocated only a limited number of studies into *de facto* statistical analysis (3 and 2, respectively) [7–8] despite of a relatively large number of selected publications. As confusing results, HA-coated cementless prostheses were compared with cemented and not with other porous-coated or non-coated cementless (uncemented) prostheses in 4 meta-analyses [3,13,14,15].

Therefore, the aim of our study was to update current knowledge and compare up-to-date data on the quality of fixation in TKA implants under two conditions: with HA-coated cementless prosthesis and with uncemented or cemented fixation. The primary outcome was MTPM of the tibial stem determined by radiostereometrical analysis (RSA). The secondary endpoints were clinical outcomes including the KSS and the KFS.

## Materials and methods

This study is reported in accordance with the PRISMA 2009 (Preferred Reporting Items in Systematic Reviews and Meta-Analysis) statement (**S1 Table**) [16]. The review protocol was registered with the National Institute for Health Research PROSPERO system under registration number CRD42019129619.

### Search

A systematic literature search was performed using EMBASE, MEDLINE, Scopus and CENTRAL. The query was designed based on Medical Subject Headings (MeSH) terms combined with various free-text terms for hydroxyapatite and uncemented or cemented prosthesis and total knee arthroplasty. No language limitation was applied **(S1 Fig)**. The date of final literature search was May 31^th^, 2019.

### Selection and eligibility criteria

**Fig 1:**
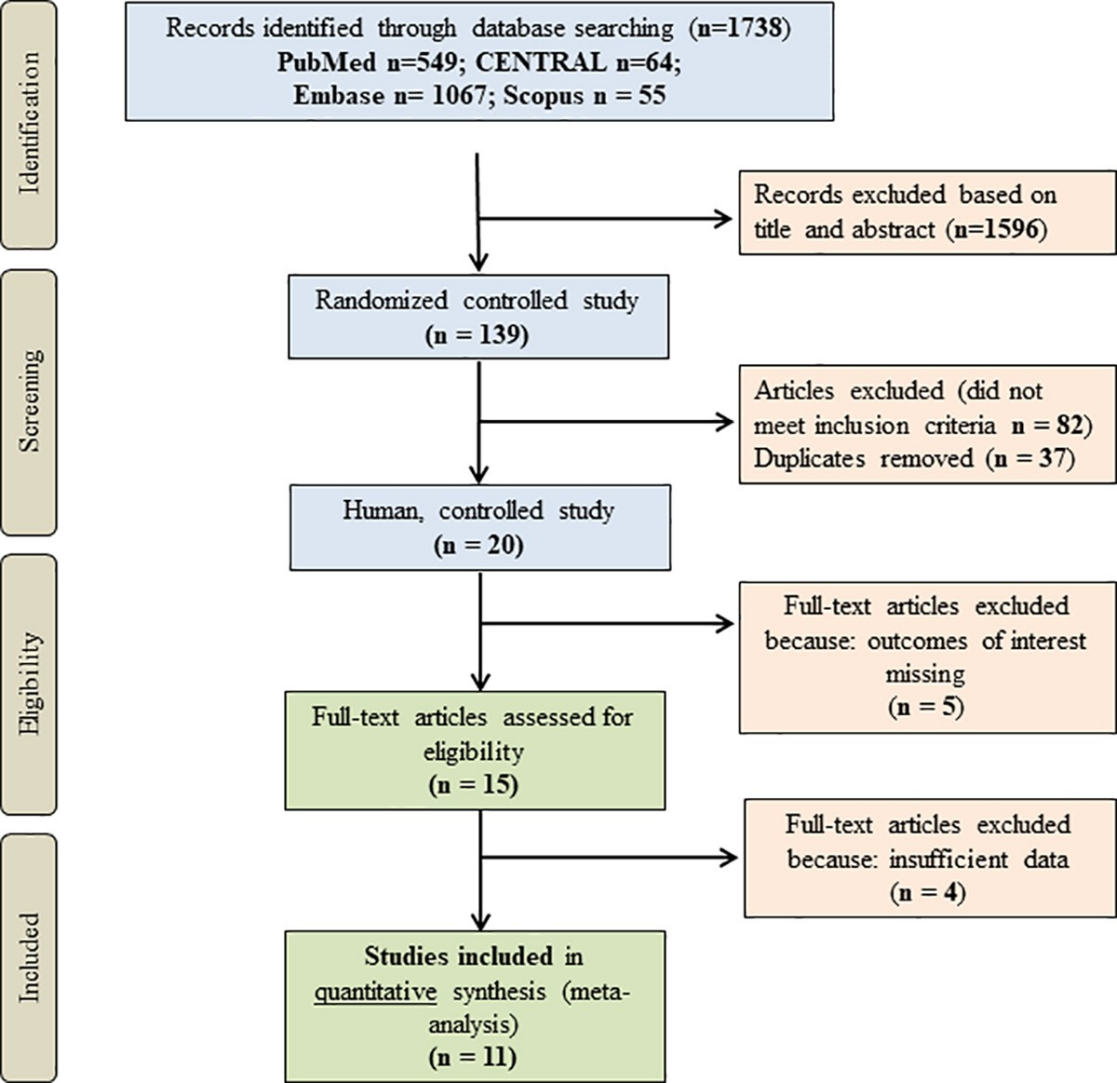
Flowchart of the meta-analysis.

Inclusion criteria specified any RCTs comparing the radiological and clinical outcomes of HA-coated tibial stem with those uncemented or cemented stems for primary TKA implants. Reoperations (revision prostheses), hybrid fixation, unicompartmental knee arthroplasty, non-clinical and uncontrolled studies were excluded. RCTs missing outcomes of our study were also excluded. Two authors (T.H. and E.B.) reviewed all studies upon the search strategy and controversies were resolved by discussion with a third author (P.H.). Full-text versions of potentially relevant studies were evaluated for inclusion using an eligibility pro forma screening document that was based on pre-specified criteria. At the end of literature search, 11 RCTs involving 902 patients were enrolled to analysis (**Fig 1**).

### Outcomes

The primary outcome was the MTPM of the tibial stem. MTPM is determined by RSA using the UMRSA software (RSA Biomedical, Umeå, Sweden) according to guideline [17] and is defined clearly in the articles as the total three-dimensional vector displacement of the marker to the greatest motion.

When useful data were presented in graphic plots, we quantified them by using open source PlotDigitizer for Windows software (Version 2.6.8, Joseph A. Huwaldt). Where mean with standard deviation was not reported, they were estimated from median, interquartiles and range by using the method of Xiang Wan [18]. The median (range) was transformed to mean±standard deviation (SD). Disagreements were resolved by discussion with a senior author or by contacting the corresponding author.

The secondary outcomes were validated using scoring systems including KSS and KFS referring to the function of the implant in everyday life. The KSS evaluates the clinical profile with regards to pain intensity, range of motion and stability, flexion deformities, contractures and poor alignment. In contrast, KFS considers only walking distance and stair climbing with deduction for walking aids.

### Data extraction and risk of bias assessment

**Fig 2:**
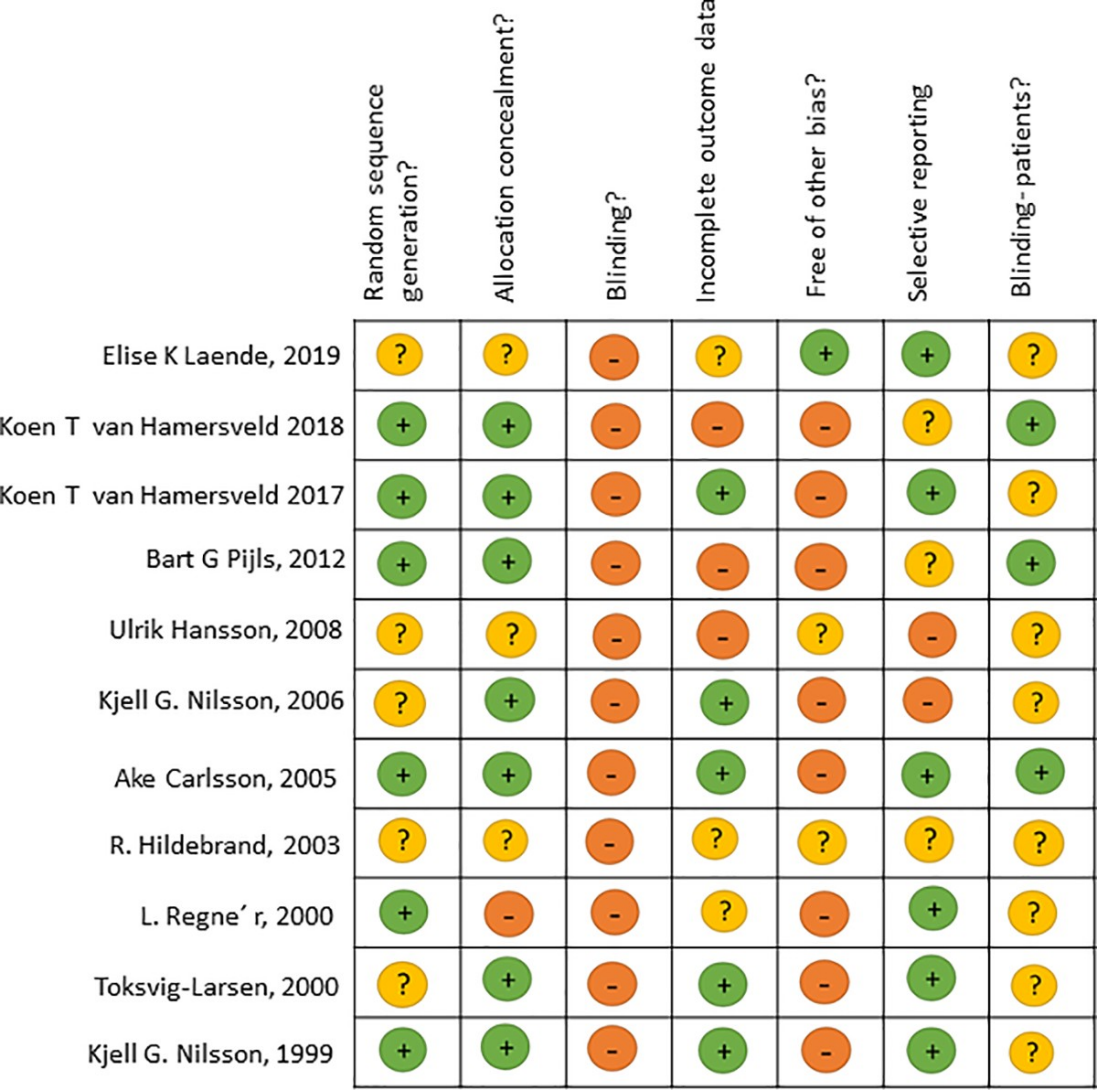
Risk of bias—Review of authors’ judgments about each risk of bias item for each study included.

We used the Cochrane risk-of-bias tool to assess the risk of bias for each study (**Fig 2**) [19]. Demographic, quality, and outcome data were extracted independently into Microsoft Excel by two authors (T.H. and E.B.). Data were taken from all articles describing the studies. Any questions in data extraction were settled by discussion with a third author.

### Quality of evidence

In order to estimate the quality of evidence on the outcomes in our meta-analysis, we have used the Grading of Recommendations Assessment, Development, and Evaluation (GRADE) approach (**S4 Table**).

### Statistical analysis

The statistical analysis of this study was performed by a dedicated statistician (L.H.) using Stata 15 SE (Stata Corp) pooled weighted mean difference (WMD) with 95% CI was calculated for continuous outcomes. Random effect model was was applied to all analyses with DerSimonian-Laird estimation. Statistical heterogeneity was analysed using the I^2^ and the chi-square statistic to gain probability-values; I^2^ represents the magnitude of the heterogeneity (moderate: 30–60%, substantial: 50–90%, considerable: 75–100%).

Publication bias was evaluated by visual inspection of the funnel plot, and the presence of this bias was considered in the case of an asymmetrical rather than a symmetrical graph. These funnel plots were automatically generated by the Stata software using the effect size and the standard error of the effect size for each study. Due to the low number of included studies per analysis (less than 10), the conditions of the Egger’s test were not met. We performed trial sequential analysis (TSA) for primary outcomes. We used TSA program version 0.9 beta (available from www.ctu.dk/tsa) to determine whether further randomized trials are needed in this investigation (**S2 Fig**).

## Results

Eleven RCTs were included in quantitative synthesis, in which TKA implants with a HA-coated tibial stem was compared to other tibial fixations (cemented and uncemented prosthesis). All trials were homogenous with respect to demographic characteristics. (**Table 1**).

**Table 1:** Characteristics of the studies included.

Author, Year	Design	Country	Recruitment period	Patients’ characteristics					
				Patients	N^0^ of knees	Age (y)		Gender (male%)	BMI
						Mean	SD		Mean
**Carlsson** **2005** [4]	prospective randomized	Sweden	1992–1995	30	72	72,6	6	21,3	ND
**Laende** **2019** [14]	prospective randomized	Australia	2002–2015	ND	360	65	7,8	61	31,6
**Pijls** **2012** [15]	prospective randomized	Sweden	ND	ND	68	62	ND	18,3	26,5
**Hildebrand 2003** [20]	prospective randomized	Germany	1992–1993	48	27	70,7	ND	ND	ND
**Regne´ r** **2000** [21]	prospective randomized	Sweden	ND	68	51	66,5	ND	16	ND
**Nilsson** **1999** [22]	prospective randomized	Sweden	1991–1992	53	27	67	ND	17	ND
**Nilsson** **2006** [23]	prospective randomized	Sweden	1997–2003	85	69	55,7	ND	62	ND
**Toksvig** **2000** [24]	prospective randomized	Sweden	ND	60	62	71	ND	ND	ND
**Hansson** **2008** [25]	prospective randomized	Sweden	1997–1999	60	49	ND	ND	ND	ND
**Hamersveld 2018** [26]	prospective randomized	Sweden	2007–2008	58	25	66	7,4	17,3	ND
**Hamersveld 2017** [27]	prospective randomized	Sweden	2009–2010	60	60	66,2	7,2	16	28,3

### Radiological outcome

**Fig 3:**
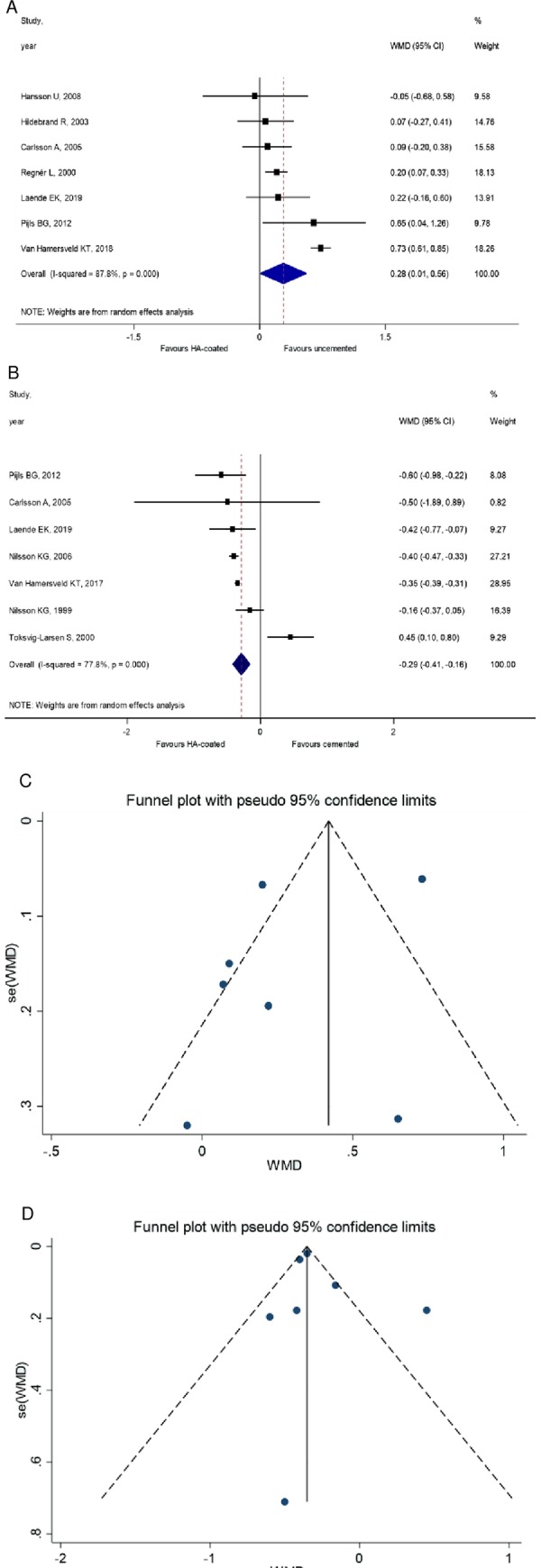
**A** MTPM analysis of the cemented and HA-coated cementless group. The value of cemented MTPM lesser than HA-coated cementless group. **B** MTPM analysis of uncemented vs. HA-coated cementless group. The MTPM values of uncemented prostheses are significantly higher than HA-coated. **C** Funnel plot 2 years follow-up; HA-coated cementless vs. uncemented group. **D** Funnel plot 2 years follow-up; HA-coated cementless vs. cemented group.

The MTPM of the tibial stem at 2 years is the primary outcome in this analysis. If the MTPM exceeds 0.2 mm, the prosthesis is classified as unstable, which greatly increases the likelihood of other complications such as aseptic loosening. If the MTPM is less than 0.2 mm, the prosthesis can be considering as stable in a long run [28]. Thirteen studies were enrolled to the MTPM analysis. The analysis showed that the MTPM values of the HA-coated cementless stems are significantly lower than that of the uncemented stems (WMD = 0.28, 95% CI: 0.01–0.56, *P* = 0.045) (**Fig 3A**).

When HA-coated implants were compared to cemented prostheses, the latter displayed lower MTPM (WMD = -0.29, 95% CI: -0.41 to 0.16, *P* < 0.001) (**Fig 3B**).

(**Fig 3C and 3D**). In these two plots show the funnel plot, but we couldn't run Egger's test on it.

### Clinical outcomes

**Fig 4:**
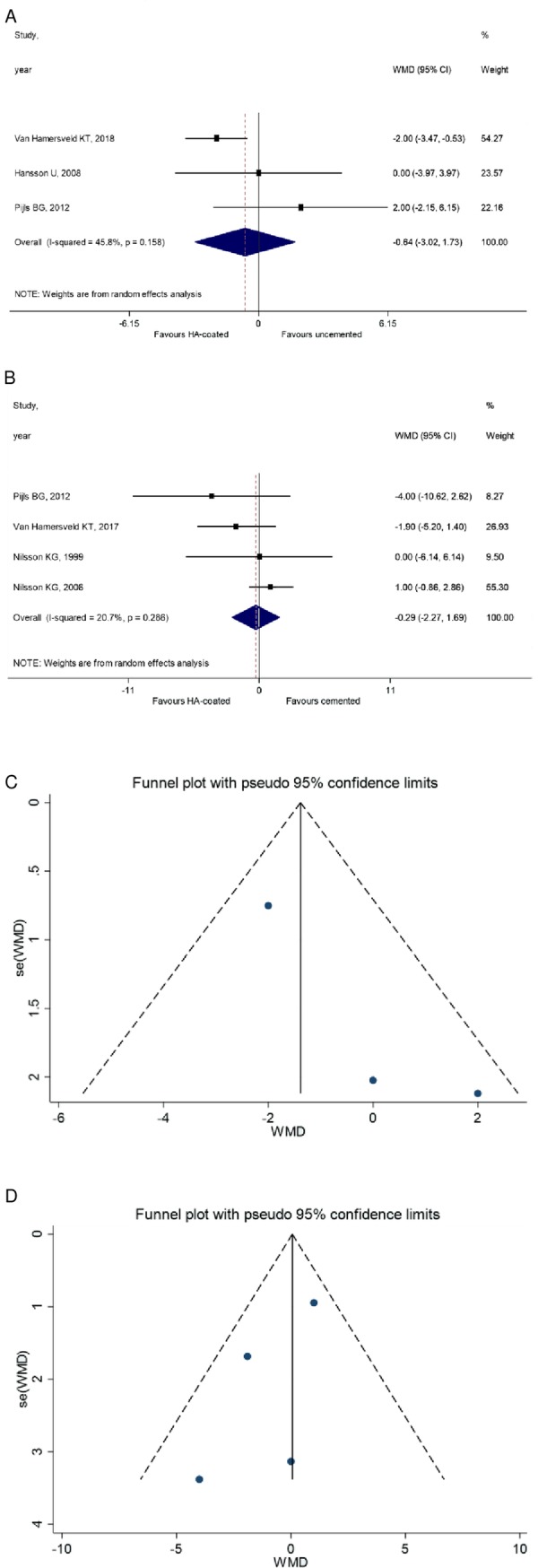
**A** KSS analysis 2 years follow-up; HA-coated. cementless vs uncemented. The value of the uncemented is lesser than of HA-coated cementless group. **B** KSS of the HA-coated cementless vs. cemented group. The value of cemented KSS did not differ significantly from that of HA-coated. **C** Funnel plot 2 years follow-up; HA-coated cementless vs. uncemented group. **D** Funnel plot 2 years follow-up; HA-coated cementless vs. cemented group.

**Fig 5:**
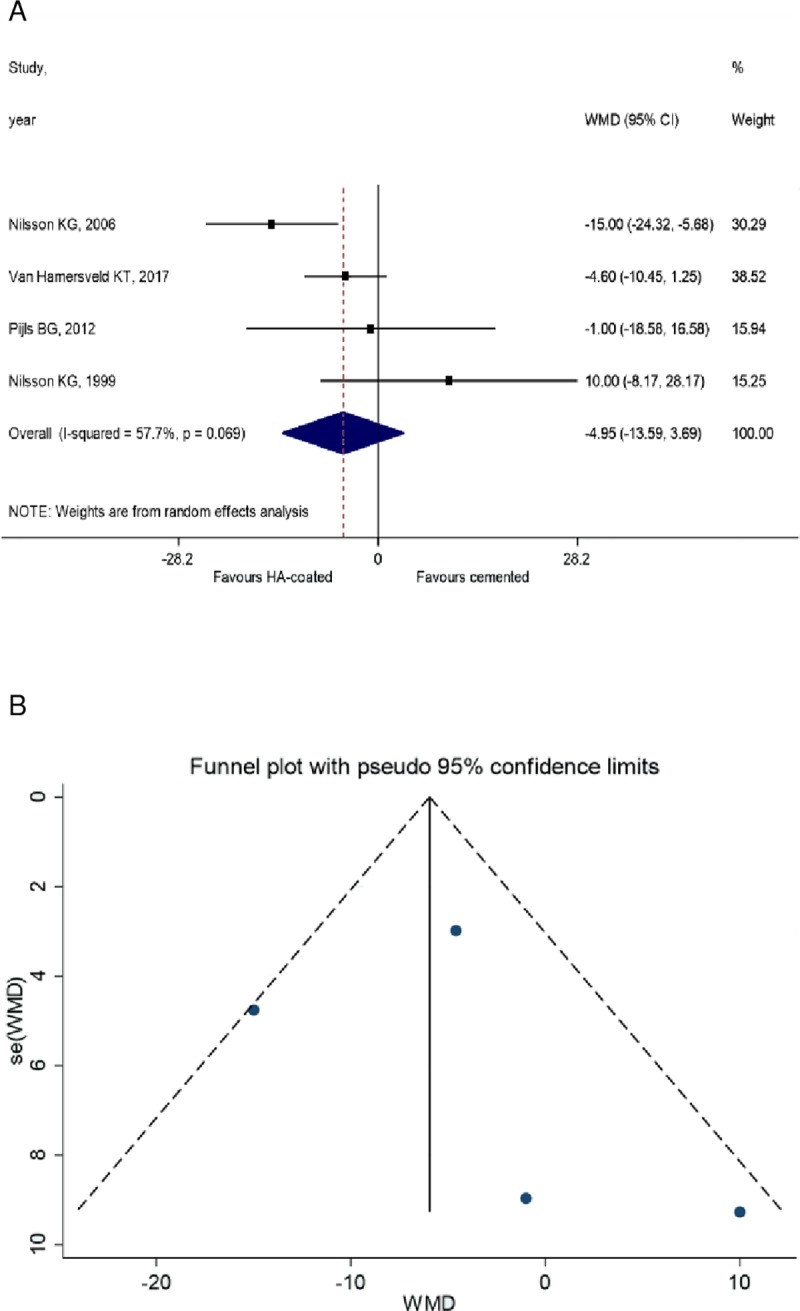
**A** KFS value of the cemented and the HA-coated cementless group. The value of cemented is not significantly different from the HA-coated cementless group. **B** Funnel plot 2 years follow-up; HA-coated cementless vs. uncemented group.

The secondary outcomes were KSS and KFS. Four RCTs were enrolled to the analysis. The analysis showed that KSS of HA-coated cementless prostheses is not significantly different from that of the uncemented group (WMD = -0.64, 95% CI: -3.02–1.73, *P* = 0.596) (**Fig 4A**);

Of interest, there was no statistically significant difference between the KSS of HA-coated cementless and cemented prosthesis (WMD = -0.29, 95% CI: -2.27 to 1.69, *P* = 0.775) (**Fig 4B**). Similar results could be obtained from the analysis of KFS, however, have limited value due to the lack of the comparison between HA-coated and uncemented groups. As such, no significant difference could be observed between HA-coated cementless and cemented implants (WMD = -4.95, 95% CI: -13.59 to 3.69 *P* = 0.069). However, comparison between HA-coated and uncemented groups was not performed due to the low number of studies in the uncemented group. (**Fig 5A**). (**Figs 4C, 4D and 5B)** KSS data are shown on the funnel plot, but unfortunately we couldn't run Egger's test on it.

## Discussion

This study reviews the current evidence on and updates knowledge of the use of HA-coated tibial stem for primary TKA implants. The treatment groups were homogenous in terms of characteristics of patients, thus the prediction of primary and secondary outcomes (i.e. MTPM and KSS and KFS) was likely independent from individual risk variables, patient selection or the overall severity of osteoporosis at prosthesis implantation. Direct meta-analysis comparison was made and the sample size of included trials was large enough to provide good evidence that HA-coating yields better stability than other, uncemented prostheses. However, cement fixation of prostheses stems still performs greater anchorage against migration. More importantly, the HA-coating is not outperformed by cemented prosthesis in providing good functional outcome with regards to pain intensity, range of motion and walking distance.

The survival probability of the stems is often cited in the literature as predictor of prosthesis outcome. However, the TKA implants outcomes are generally good with a mean survivorship rate (or projected rates) of 95% or more at 10 years. Hence, this parameter is less sensitive to evaluate the quality of stem fixation, than radiological results [29]. The migration analysis with RSA is a standardized and objective method with low susceptibility to different interpretations [17]. This technique allows movements between the implant and host bone measured with an accuracy of 0.2 mm [28,30]. As a primary outcome of our study, the migration pattern of the prosthesis stems was determined as the maximum total point motion (MTPM) of the tibial stem measured by RSA. The MTPM value is the unit of measurement for the largest 3D migration of any point on the prosthesis surface. The migration pattern was defined as at least 2 postoperative follow-up moments within the first 2 years of follow-up [28]. MTPM mainly depends on mechanical factors such as the bone-implant interface or different biological reactions at the implant-bone interface therefore is a reliable parameter to assess the added value of HA-coating in implant surface.

RCTs in our meta-analysis have demonstrated lower incidence of MTPM with HA–coated implants when compared to other non-cemented stems, except one trial [31]. As for the comparison of HA-coated cementless and cemented group, the overall rates of MTPM were very low in the cemented group and displayed lower incidence than HA-coated cementless prosthesis. It is contradictory with a recent meta-analysis of Voight and his coworkers, which demonstrated that use of HA provide the best long-term stability of implants. However, they failed to eliminate potential selection bias because of enrolled studies with hybrid fixation [7]. Another confounding factor was that HA-coated cementless fixation was compared to an inhomogenic group of cemented and uncoated or other-coated cementless fixations. Some other meta-analyses have demonstrated equal stability by using cemented and cementless implants [5,6,8]. Registry data support that risk of revision rate is significantly higher in uncemented TKA implants in comparison with cemented prosthesis, and the main reason is aseptic loosening [10–11]. The contradictory conclusions derived from these data can be explained by the selection bias of database analysis.

Clinical outcomes, the KSS and KFS in our meta-analysis demonstrated equal functionality of HA-coated cementless and cemented implants. These scoring systems are validated and responsive methods for assessing objective and subjective outcomes after TKA implants. KSS is a weighted score which regards to pain intensity, range of motion, stability and flexion deformities, contractures and poor alignment. The KFS considers mobility parameters of the patient such as the walking distance and stair climbing with deduction for walking aids. In spite of the predictive value of radiological stability, a recent meta-analysis has revealed the differences between postoperative radiological and clinical performance of TKA implants at the same time [5]. Our result is consistent with this previous finding.

The final outcome of TKA implants can also be linked to factors such as the prosthesis type and the risk of developing certain complications of the patient. Early generation of cementless prosthesis demonstrated poor results due to the suboptimal design of the implants [32]. In order to exclude bias derived from the different design of prosthesis types, we enrolled studies comparing HA-coated prostheses with other prostheses from the same uncemented or cemented series of the manufacturer (**S2 Table**).

This study has some limitations. Low survival probability and revision rate of the stems are often cited in the literature as predictor of poor outcome and these factors were not considered in the selected trials. Different trials presented some alterations concerning the operative procedure, whose impact on the outcomes were not evaluated. Besides, comparison of KFS in HA-coated and uncemented groups would have limited value due to the low number of studies in the uncemented group. The included RCTs were homogenous with regard to patient parameters, which, on the one hand provided possibility to exclude selection bias, but on the other hand, the effects of medication, physiotherapy, activity level or systemic diseases (e.g. osteoporosis or osteopenia) could not be evaluated. It would be also important to compare the individual types of cementless knee prosthesis and the outcome of their implantation [33].

## Conclusion

In conclusion, this review provides the best available evidence that HA-coated cementless prosthesis outperforms other cementless prostheses both in respect to stability and functionality. Cemented fixation of prostheses provide the best stability in a 2-year follow-up, however, functional results are not superior to HA-coated cementless fixation. Based on these results, HA-coated cementless TKA implants is a recommended option for treating end-stage arthritis of the knee, and clinicians consider together with patients the factors associated with the risk of revision when choosing the most appropriate procedure.

## Supporting information

S1 Table PRISMA checklist for preferred reporting items for systematic reviews and meta-analyses. (PDF)

S2 Table Type of prosthesis. (PDF)

S3 Table KSS and KFS data together with the MTPM values. (PDF)

S4 Table Quality of evidence. (PDF)

S1 Fig Search strategy. (PDF)

S2 Fig Trial sequential analysis of the primary outcomes. A: The cumulative z-curve surpassed the conventional boundary for statistical significance. However, none of the trial sequential monitoring boundaries have been surpassed in the TSA. Therefore, the result is inconclusive, the required information size (1723) has not yet been achieved. B: The cumulative z-curve crossed both the conventional boundary and the trial sequential monitoring boundary, and the required information size has been achieved. There is no need to include further studies to confirm the significant result.
(PDF)

## List of abbreviations

BMI: body mass index
CI: confidence interval
HA: hydroxyapatite
KFS: knee society function score
KSS: knee society knee score
MTPM: maximum total point motion
RCT: randomized controlled trials
RSA: radiosterometrical analysis
TKA: total knee arthroplasty
TSA: trial sequential analysis
WMD: weighted mean difference
