# Supplementary material for: Hydroxyapatite-coated implants provide better fixation in total knee arthroplasty. A meta-analysis of randomized controlled trials
Source: PLoS One. 2020 May 12;15(5):e0232378. doi: 10.1371/journal.pone.0232378 (PMC7217427; doi:10.1371/journal.pone.0232378)
Supplement: S2 Table — (PDF) [file pone.0232378.s002.pdf]

First Author	Groups	Prosthesis type
Laende (2019)	HA-coated	Triathlon (Stryker)
	uncemented	Advance Biofoam (Wright Medical technology inc.) Trabecular Metal Monoblock (Zimmer) Trabecular Metal Modular (Zimmer)
	cemented	Advance (Wright Medical Technoloy Inc.) NexGen (Zimmer) Triathlon (Stryker)
Hamersveld (2018)	HA-coated	Triathlon (Stryker)
	uncemented	Triathlon-(Stryker)
Hamersveld (2017)	HA-coated	Triathlon (Stryker)
	cemented	Triathlon (Stryker)
Pijls (2012)	HA-coated	Interax (Howmedica)
	uncemented	Interax (Howmedica)
	cemented	Interax (Howmedica)
Hansson (2008)	HA-coated	Duracon (Stryker)
	uncemented	Duracon (Stryker)
Nilsson (2008)	Ha-coated	Profix (Smith & Nephew)
	cemented	Profix (Smith & Nephew)
Carlsson (2005)	HA-coated	CAM Implant (Camceram)
	uncemented	Press-fit Condylar Modular-PFC (Johnson & Johnson)
	cemented	Press-fit Condylar modular-PFC (Johnson & Johnson)
Hildebrand (2003)	HA-coated	Typ MG-II
	uncemented	Typ MG-II
Regne´ r (2000)	HA-coated	Freeman- Samuelson
	uncemented	Miller- Galante II
Toksvig (2000)	Ha-coated	Duracon CoCr
	cemented	Osteonic 7000 CoCr (Osteonics)
Nilsson (1999)	HA-coated	Tricon II (Smith & Nephew)
	cemented	Tricon II (Smith & Nephew)

Supplementary Table 2. *Type of prosthesis*
