# Supplementary material for: Hydroxyapatite-coated implants provide better fixation in total knee arthroplasty. A meta-analysis of randomized controlled trials
Source: PLoS One. 2020 May 12;15(5):e0232378. doi: 10.1371/journal.pone.0232378 (PMC7217427; doi:10.1371/journal.pone.0232378)
Supplement: S3 Table — (PDF) [file pone.0232378.s003.pdf]

Publication data				Clinical data												
First Author	Year	Groups	N^0 of knees	MTPM (maximum total point motion) mm					Knee Score				Knee Function Score			
				final outcome (2 year ≤)					final outcome Mean (1 year ≤)	final outcome Range (2 year ≤)			final outcome Mean (1 year ≤)	final outcome Range (2 year ≤)		
				Mean	SD	SE	CI			SD	CI			SD	CI	
							Min	Max			Min	Max			Min	Max
Elise K Laende	2019	HA-coated	32	0,84	0,19											
		Uncemented, porous coated	138	1,57	0,39											
		Cemented	222	0,47	0,19											
Koen T Van Hamersveld	2018	HA-coated	13	0,9			0,72	1,19	97		94	100	86		78	94
		Uncemented, porous coated	12	1,7			1,41	2,08	94		86	100	84		76	91
K. T Van Hamersveld	2017	HA-coated	30	0,97			0,81	1,15	91,2	13,6			86,4	20,9		
		Cemented	30	0,62			0,49	0,76	94,3	11,7			90	12,8		
Bart G Pijls	2012	HA-coated	24	1,1	0,95				85	7	80	90	46	33	23	69
		Uncemented, porous coated	20	1,75	1,1				87	7	81	92	42	32	18	67
		Cemented	24	0,5	0,15				81	15	71	91	45	29	25	65
Ulrik Hansson	2008	HA-coated	24	1,75		0,25			93		75	100				
		Uncemented, porous coated	25	1,7		0,2			93		66	100				
Kjell G. Nilsson	2006	Ha coated	34	1,22	0,21				94		77	100	100		40	100
		Cemented	35	0,82	0,03				95		95	100	85		0	100
Ake Carlsson	2005	HA-coated	27	1,05		0,12			86		33	100	65		0	100
		Uncemented, porous coated	19	1,14		0,09			86		33	100	65		0	100

		Cemented	26	0,55		0,7			86		33	100	65		0	100
R. Hildebrand	2003	HA-coated	14	0,66	0,38											
		Uncemented, porous coated	13	0,73	0,5											
L. Regne´r	2000	HA-coated	25	0,6		0,03										
		Uncemented, porous coated	26	0,8		0,06										
Toksvig-Larsen S	2000	HA-coated	32	1,2	0,7											
		Cemented	30	1,65	0,7											
Kjell G. Nilsson	1999	HA-coated	13	0,58		0,09			93		74	99	80		40	100
		Cemented	14	0,42		0,06			93		69	99	90		0	100

Supplementary Table 3. KSS and KFS data together with the MTPM values
