# Supplementary material for: Hydroxyapatite-coated implants provide better fixation in total knee arthroplasty. A meta-analysis of randomized controlled trials
Source: PLoS One. 2020 May 12;15(5):e0232378. doi: 10.1371/journal.pone.0232378 (PMC7217427; doi:10.1371/journal.pone.0232378)
Supplement: S4 Table — (PDF) [file pone.0232378.s004.pdf]

Supplementary Table 4. Quality of evidence

Should HA-coated vs. cemented be used for patients of TKA?

HA-coated compared to cemented for patients of TKA

Certainty assessment							Summary of findings					Importance
							№ of patients		Effect		Certainty	
№ of studies	Study design	Risk of bias	Inconsistency	Indirectness	Imprecision	Other considerations	HA-coated	Cemented	Relative (95% CI)	Absolute (95% CI)		
**Maximum total point motion (follow up: 2 years)**												
11	randomised trials	serious	not serious	not serious	not serious	none	268	381	-	WMD **0.29 lower** (0.41 lower to 1.16 higher)	⨁⨁⨁◯ MODERATE	IMPORTANT
**Knee society knee score (follow up: 2 years)**												
4	randomised trials	serious	not serious	not serious	not serious	none	101	103	-	WMD **0.29 lower** (2.27 lower to 1.69 higher)	⨁⨁⨁◯ MODERATE	IMPORTANT
**Knee function knee score (follow up: 2 years)**												
3	randomised trials	serious	not serious	not serious	not serious	none	61	80	-	WMD **4.95 lower** (13.59 lower to 3.69 higher)	⨁⨁⨁◯ MODERATE	IMPORTANT

Should Ha-coated vs. cementless be used for patients of TKA?

Ha-coated compared to cementless for patients of TKA

Certainty assessment							Summary of findings					Importance
							№ of patients		Effect		Certainty	
№ of studies	Study design	Risk of bias	Inconsistency	Indirectness	Imprecision	Other considerations	Ha-coated	Cementless	Relative (95% CI)	Absolute (95% CI)		
**Maximum total point motion (follow up: 2 years)**												
11	randomised trials	serious	not serious	not serious	not serious	none	268	253	-	WMD **0.28 lower** (0.01 higher to 0.56 higher)	⨁⨁⨁◯ MODERATE	IMPORTANT
**Knee society knee score (follow up: 2 years)**												
4	randomised trials	serious	not serious	not serious	not serious	none	61	57	-	WMD **0.64 lower** (3.02 lower to 1.73 higher)	⨁⨁⨁◯ MODERATE	IMPORTANT
