# Supplementary material for: Hydroxyapatite-coated implants provide better fixation in total knee arthroplasty. A meta-analysis of randomized controlled trials
Source: PLoS One. 2020 May 12;15(5):e0232378. doi: 10.1371/journal.pone.0232378 (PMC7217427; doi:10.1371/journal.pone.0232378)
Supplement: S1 Fig — (PDF) [file pone.0232378.s005.pdf]

Search strategy

Search results May 31th, 2019

CENTRAL (Cochrane Library)

(('apatite'/exp OR apatite OR 'hydroxyapatite'/exp OR hydroxyapatite OR periapatite OR 'peri-apatite-coated' OR 'periapatite-coated' OR '(pa)-coated' OR 'hydroxyapatite-coated' OR '(ha)-coated' OR 'uncemented cruciate retaining' OR 'cemented cruciate retaining' OR 'titanium-coated' OR uncoated OR 'cementless tka') AND ('knee replacement'/exp OR 'knee replacement' OR 'kr' OR 'total knee replacement'/exp OR 'total knee replacement' OR 'tkr' OR 'knee arthroplasty'/exp OR 'knee arthroplasty' OR 'ka' OR 'total knee arthroplasty'/exp OR 'total knee arthroplasty' OR 'tka')) AND 'randomized controlled trial'/de

Embase.com

(('apatite'/exp OR apatite OR 'hydroxyapatite'/exp OR hydroxyapatite OR periapatite OR 'peri-apatite-coated' OR 'periapatite-coated' OR '(pa)-coated' OR 'hydroxyapatite-coated' OR '(ha)-coated' OR 'uncemented cruciate retaining' OR 'cemented cruciate retaining' OR 'titanium-coated' OR uncoated OR 'cementless tka') AND ('knee replacement'/exp OR 'knee replacement' OR 'kr' OR 'total knee replacement'/exp OR 'total knee replacement' OR 'tkr' OR 'knee arthroplasty'/exp OR 'knee arthroplasty' OR 'ka' OR 'total knee arthroplasty'/exp OR 'total knee arthroplasty' OR 'tka')) AND 'randomized controlled trial'/de

PubMed.com

Apatites"[Mesh]) AND "Arthroplasty, Replacement, Knee"[Mesh]; filtration: clinical trials

Scopus.com

(('apatite'/exp OR apatite OR 'hydroxyapatite'/exp OR hydroxyapatite OR periapatite OR 'peri-apatite-coated' OR 'periapatite-coated' OR '(pa)-coated' OR 'hydroxyapatite-coated' OR '(ha)-coated' OR 'uncemented cruciate retaining' OR 'cemented cruciate retaining' OR 'titanium-coated' OR uncoated OR 'cementless tka') AND ('knee replacement'/exp OR 'knee replacement' OR 'kr' OR 'total knee replacement'/exp OR 'total knee replacement' OR 'tkr' OR 'knee arthroplasty'/exp OR 'knee arthroplasty' OR 'ka' OR 'total knee arthroplasty'/exp OR 'total knee arthroplasty' OR 'tka')) AND 'randomized controlled trial'/de
