# Supplementary material for: Hydroxyapatite-coated implants provide better fixation in total knee arthroplasty. A meta-analysis of randomized controlled trials
Source: PLoS One. 2020 May 12;15(5):e0232378. doi: 10.1371/journal.pone.0232378 (PMC7217427; doi:10.1371/journal.pone.0232378)
Supplement: S2 Fig — A: The cumulative z-curve surpassed the conventional boundary for statistical significance. However, none of the trial sequential monitoring boundaries have been surpassed in the TSA. Therefore, the result is inconclusive, the required information size (1723) has not yet been achieved. B: The cumulative z-curve crossed both the conventional boundary and the trial sequential monitoring boundary, and the required information size has been achieved. There is no need to include further studies to confirm the significant result. (PDF) [file pone.0232378.s006.pdf]

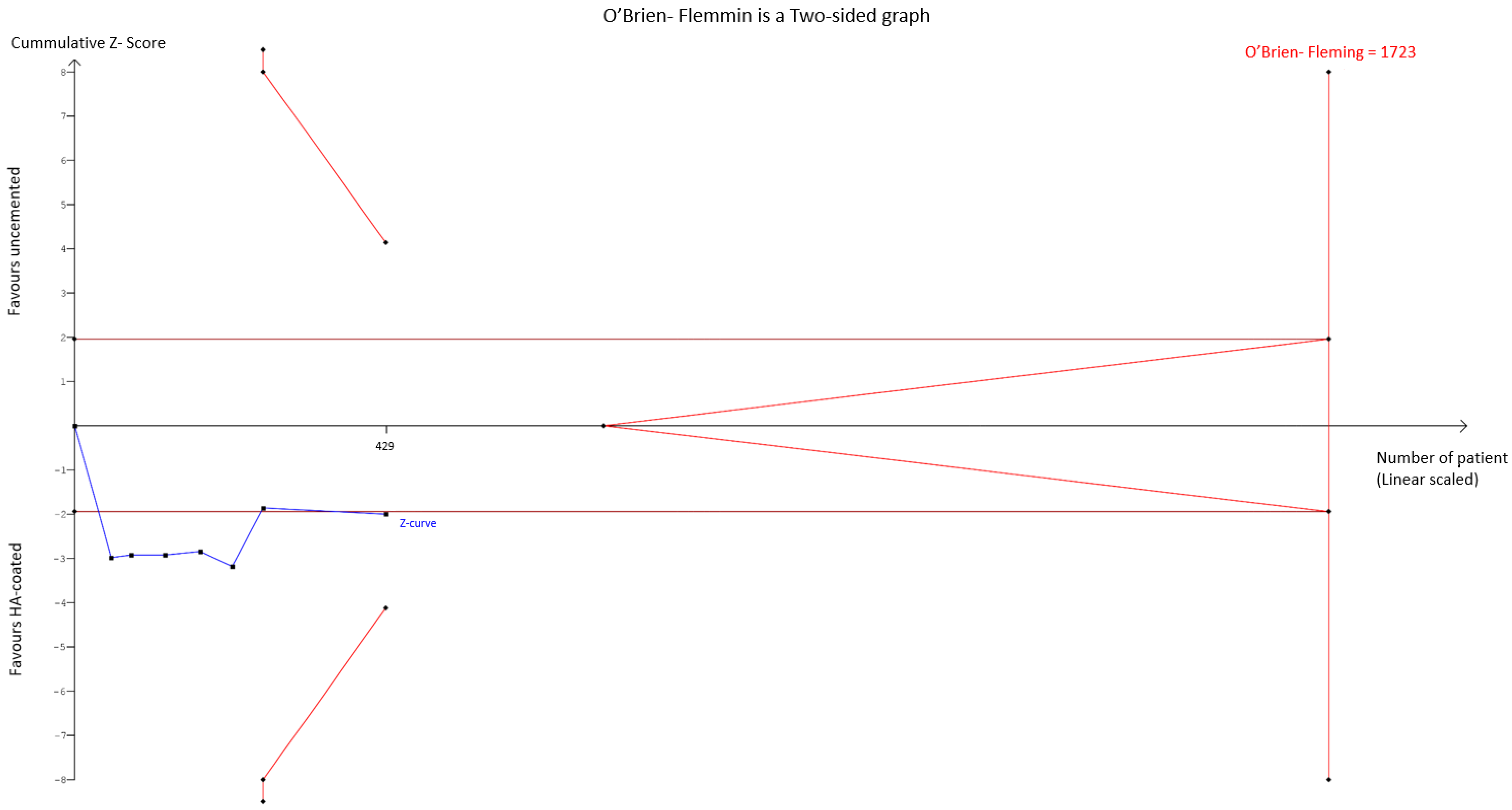

A: The cumulative z-curve surpassed the conventional boundary for statistical significance. However, none of the trial sequential monitoring boundaries have been surpassed in the TSA. Therefore, the result is inconclusive, the required information size (1723) has not yet been achieved.

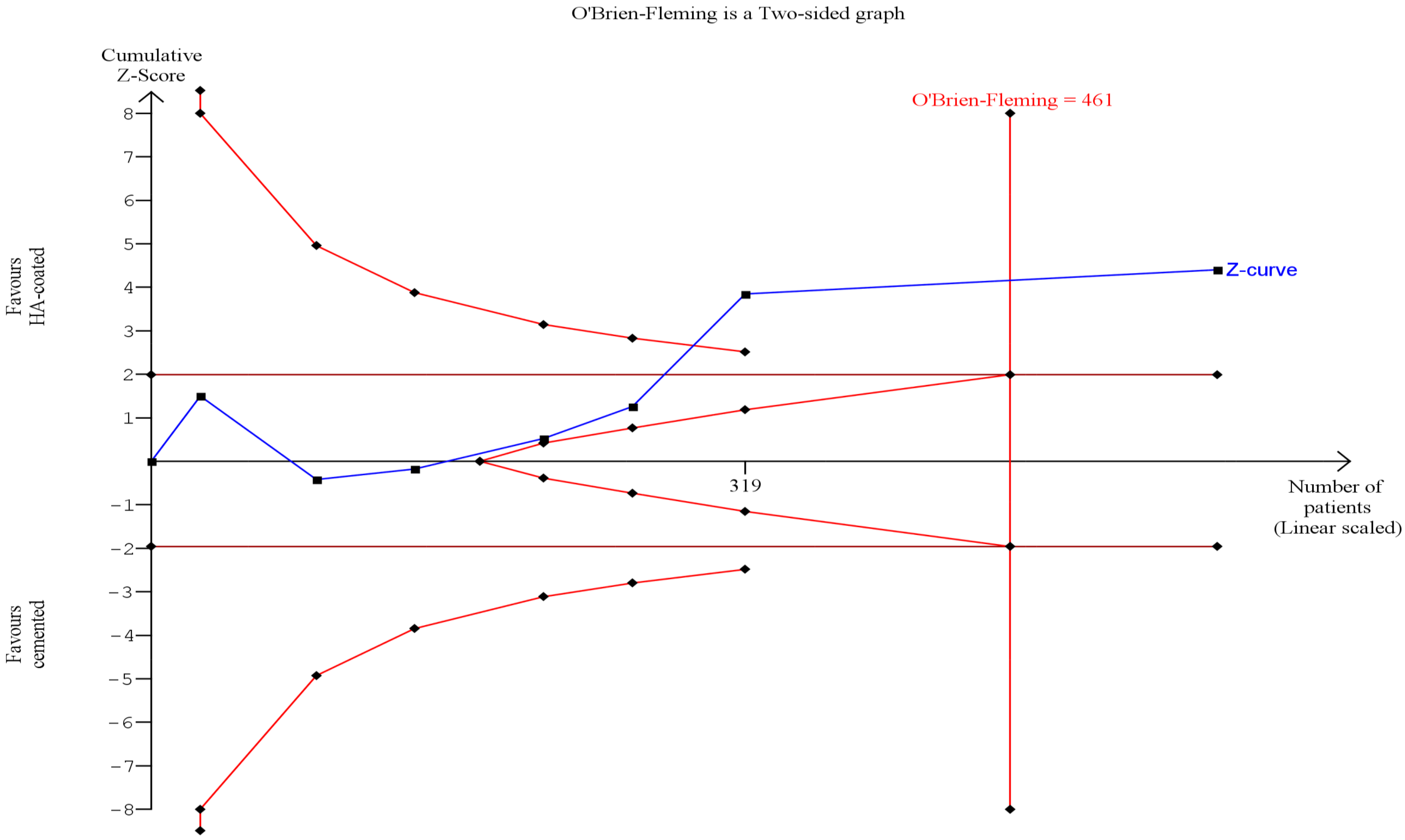

B: The cumulative z-curve crossed both the conventional boundary and the trial sequential monitoring boundary, and the required information size has been achieved. There is no need to include further studies to confirm the significant result.
